# Dietary Considerations in Autism Spectrum Disorders: The Potential Role of Protein Digestion and Microbial Putrefaction in the Gut-Brain Axis

**DOI:** 10.3389/fnut.2018.00040

**Published:** 2018-05-18

**Authors:** Megan R. Sanctuary, Jennifer N. Kain, Kathleen Angkustsiri, J. Bruce German

**Affiliations:** ^1^Department of Nutrition, University of California, Davis, Davis, CA, United States; ^2^Department of Neurobiology, Physiology and Behavior Department, University of California, Davis, Davis, CA, United States; ^3^School of Medicine, Department of Pediatrics, University of California, Davis, Sacramento, CA, United States; ^4^Department of Pediatrics, UC Davis MIND Institute, Sacramento, CA, United States; ^5^Department of Food Science and Technology, University of California, Davis, Davis, CA, United States; ^6^Foods for Health Institute, University of California, Davis, Davis, CA, United States

**Keywords:** microbiota, gastrointestinal, dietary protein, gut-brain, fragile gut, probiotics, constipation, diarrhea

## Abstract

Children with autism spectrum disorders (ASD), characterized by a range of behavioral abnormalities and social deficits, display high incidence of gastrointestinal (GI) co-morbidities including chronic constipation and diarrhea. Research is now increasingly able to characterize the “fragile gut” in these children and understand the role that impairment of specific GI functions plays in the GI symptoms associated with ASD. This mechanistic understanding is extending to the interactions between diet and ASD, including food structure and protein digestive capacity in exacerbating autistic symptoms. Children with ASD and gut co-morbidities exhibit low digestive enzyme activity, impaired gut barrier integrity and the presence of antibodies specific for dietary proteins in the peripheral circulation. These findings support the hypothesis that entry of dietary peptides from the gut lumen into the vasculature are associated with an aberrant immune response. Furthermore, a subset of children with ASD exhibit high concentrations of metabolites originating from microbial activity on proteinaceous substrates. Taken together, the combination of specific protein intakes poor digestion, gut barrier integrity, microbiota composition and function all on a background of ASD represents a phenotypic pattern. A potential consequence of this pattern of conditions is that the fragile gut of some children with ASD is at risk for GI symptoms that may be amenable to improvement with specific dietary changes. There is growing evidence that shows an association between gut dysfunction and dysbiosis and ASD symptoms. It is therefore urgent to perform more experimental and clinical research on the “fragile gut” in children with ASD in order to move toward advancements in clinical practice. Identifying those factors that are of clinical value will provide an evidence-based path to individual management and targeted solutions; from real time sensing to the design of diets with personalized protein source/processing, all to improve GI function in children with ASD.

## Introduction

Autism spectrum disorder (ASD) is characterized by varying degrees of neural dysfunction that lead to behavioral abnormalities including: stereotyped/repetitive behaviors, impairments in social interaction, lack of verbal and nonverbal language skills, insistence on routines and intense or idiosyncratic interests (1–3). ASD includes a heterogeneous range of disorders with various levels of intellectual functioning, verbal abilities, and multiple genetic etiologies. The estimated prevalence of autism in 8-year-olds in 2012 was 1:68 children with a much higher occurrence in boys (23.6 per 1,000) than in girls (5.3 per 1,000) (4). Over the past decade, the incidence rate has been steadily increasing (5). While increased awareness, concern and diagnostic criteria of ASD has expanded, some postulate that this increased prevalence cannot be entirely attributed to increased recognition and more expansive diagnostic criteria (5). There are currently no biomarkers for ASD and diagnosis is made solely on the history and observation of behavioral abnormalities using the Autism Diagnostic Interview - Revised (ADI-R) assessment (6) and the Autism Diagnostic Observational Schedule (ADOS) (7). Treatment of ASD symptoms focus on augmenting the obvious behavioral abnormalities with pharmacologic, cognitive, social, and communication therapies.

There is active research into the mechanisms that cause different autism phenotypes, including studies of genetic markers, structural, and functional brain abnormalities, immune dysfunction, gut microbial dysbiosis, sensitivity to environmental toxin exposure, and oxidative stress (8–13). However, gastrointestinal dysfunction is common in children with ASD (14) yet overlooked as unrelated to the disease process, or viewed as a disorder that simply co-occurs with the autistic symptoms. There is a strong correlation between the presence of gastrointestinal symptoms and autism severity (15) as ASD patients with gastrointestinal symptoms display higher measures of irritability, anxiety and social withdrawal (16). Gut dysfunction in children with ASD deserves further investigation, especially given the potential for intervention. Gut function is now recognized as highly variable, even within the normal population; many disease processes occurring at distant body sites have been found to have origins in gut, microbial composition and metabolism (17).

The concept of the “fragile gut” is proposed to be involved in a subset of children with ASD and that impaired gastrointestinal (GI) function, including deficiency in protein digestive capacity, is central to the GI symptoms associated with ASD and may exacerbate behavioral symptoms in ASD.

### Gastrointestinal and immune dysfunction in children with ASD

Gastrointestinal co-morbidities are common in children with ASD and have been reported to affect 9–91% of patients (18–21) with the prevalence of symptoms four times greater than in children without ASD (14). This unusually broad range is due to the variability in symptoms included in diagnosis as well as the limited number of studies assessing the prevalence of gastrointestinal symptoms. In addition, these symptoms are often underreported because many of these children are nonverbal or have difficulty with communication, and many parents have difficulty recognizing gastrointestinal symptoms in their children. Even so, the most commonly reported symptoms include chronic constipation, diarrhea, and abdominal pain (14, 20, 21). Food allergies are also more common in children with ASD than age-matched controls (22) and may also be under-diagnosed due to the similar reasons (2). Certain foods appear to trigger both gastrointestinal and behavioral symptoms in children with ASD (18). To date, the poor reactions to particular foods experienced by these children has not been explicitly linked with differences in gut microbiota, immune function and digestive capacity in children with ASD relative to typically developing children.

Morphologically, there is evidence to suggest that gastrointestinal structure is altered in children with ASD. Examination of distal ileal biopsies from children with ASD reveals crypt cell proliferation, thickening of the basolateral membrane (23) of the gut epithelium and increased intestinal permeability (24, 25). These observations all suggest chronic immune activation in the gut and epithelial damage. One study found abnormally high intestinal permeability in nearly 40% of autistic patients and in 21% of their first-degree relatives whereas this excessive permeability was present in <5% of normal control subjects (24, 25). Increased permeability would contribute to heightened reactions to food components in the intestinal tract of children with ASD. Interestingly, individuals with ASD on specific protein-restricted diets (wheat and dairy free) have significantly lower intestinal permeability than those on unrestricted diets (25). Children with ASD and gastrointestinal symptoms experience unique gastrointestinal immunopathology characterized by ileal nodular lymphoid hyperplasia (26), high levels of mucosal pro-inflammatory lymphocyte infiltration with inversely low levels of regulatory lymphocyte populations (27), and colonic lesions containing high numbers of infiltrating γδ and CD8^+^ T cells with associated epithelial damage (23). Unlike inflammatory bowel diseases, there is lack of clear T_H_1 or T_H_2 skewing in ASD with the observation that IL-4, IL-2, and interferon gamma (IFN-γ) are elevated in the duodenal mucosa while IL-4 is elevated in the colon (27). Immune-mediated epithelial damage to the gut lining via serum IgG and complement C1q co-localization at the basolateral membrane (28) suggests that autoimmune mechanisms may play a role. Autoimmunity may arise out of increased immune responses to dietary proteins and potential cross reactivity to proteins in the gut or brain, which will be discussed in more detail in the following sections. Interestingly, children on specific protein-restricted diets show lower levels of activated colonic intraepithelial lymphocytes and underlying lamina propria lymphocytes (CD3^+^TNFα^+^) compared to children on unrestricted diets (27).

Immune abnormalities in ASD are not restricted to the gut and systemic immune imbalance is common. Changes in numbers and activation of macrophages, T cells, B cells, and natural killer cells have all been observed in patients with ASD (29, 30). While some studies have observed a shift from T_H_1 to T_H_2 cell type with decreased production of IL-2 and IFN-γ and increased production of IL-4 (31), others state that there is no clear cytokine polarization profile, and attempting to categorize the disease based on the traditional T_H_1/T_H_2 dichotomy may oversimplify and incorrectly characterize the immune dysfunction in children with ASD (32). Interestingly, *in vitro* stimulation of peripheral blood mononuclear cells from children with ASD with certain dietary proteins (including β-lactoglobulin and α-lactalbumin from cow's milk and gliadin from wheat but not casein cow milk protein or soy protein) resulted in elevated pro-inflammatory cytokine production when compared to healthy controls as well as non-ASD controls with non-allergic food hypersensitivity (33, 34). Together, these observations suggest that overall gut immune function is altered and food antigens may provide a pro-inflammatory stimulus in children with ASD and gastrointestinal symptoms. However, it has been unclear whether these manifestations simply co-occur with the disorder, are a result of the abnormal neural functions that characterize the disorder, or are contributing to the etiology of the disease state.

### Characterization of the “fragile gut”

Abnormal gastrointestinal functions in children with ASD include diminished digestive capacity. It is generally assumed that well-nourished, healthy children and adults who lack a history of gastrointestinal symptoms (e.g., chronic diarrhea, constipation, gastric reflux) or pancreatic insufficiency have little or no undigested or unabsorbed nutrients in the feces. The gastrointestinal tract digests and absorbs nutrients from consumed food matrices while excluding harmful bacteria and toxins. Pancreatic enzymes are produced in amounts that exceed substrate requirements allowing for complete digestion. Brush border enzymes and transporters have expression levels and activities capable of ensuring total absorption of nutrients in the small intestine (35). However, these normally successful processes may be disturbed in children with ASD and gastrointestinal symptoms.

The gut is the largest immune organ and the main site of immune system development (36). Considering that the gut immune system constantly encounters foreign material, immune imbalance can cause a lack of tolerance to dietary components and commensal microbes (37). The gastrointestinal tract is the second largest site of neurological tissue in the human body playing roles from nutrient sensing to appetite regulation (38).

For the purposes of this review a “healthy gut” is one that is able to (1) effectively extract and absorb nutrients from complex food matrices; (2) properly identify foreign materials as benign (e.g., common food substances) or harmful (e.g., pathogenic bacteria); (3) support the colonization of the gut by commensal organisms while eliminating pathogens; (4) restrict uptake or passage of potentially damaging substances out of the gut lumen through maintenance of gut barrier integrity; and (5) provide appropriate nutrient and energy status signals to other organ systems (35). All of these “healthy gut” functions are vital, and therefore, dysfunction of any one of these processes can cause or exacerbate disease throughout the body.

In this review, we will define the term “fragile gut” as a gastrointestinal tract that is basically “healthy” (i.e., lack of defined gastrointestinal disease), but is not robust to stressors, challenges, or microbial insults. Stressors include diets containing indigestible or toxic components or infectious agents. The concept is that a fragile gut influences a variety of clinical and non-clinical situations where gut dysfunction is not the explicit disease state or site, but rather a secondary problem. Examples of populations with a fragile gut include premature infants, the elderly, patients undergoing chemotherapy and children with ASD and gastrointestinal symptoms. The fragile gut phenotype in children with ASD includes a distribution of dysbiotic and pathological states. While a subset has been diagnosed as exhibiting explicit immunopathologies, others are asymptomic normally, but become symptomatic when confronted with challenges from poorly digested diets. Transient blooms of detrimental bacteria (e.g., proteobacteria) and “pulses” of microbial metabolites have been observed, as discussed below (Tables 1, 2). The causes of fragile gut remain incompletely understood. We hypothesize that the fragile gut's etiology results from a range of factors including digestive capacity, the digestibility of food ingredients consumed, genetic susceptibility as it affects gastrointestinal regulatory modulation, immune tolerance development, and gut microbial composition.

**Table 1:** Overview of studies exploring the gut microbiota in children with ASDs.

**Comparison**	**Sample site**	**Method**	**Level**	**Type**	**Change**	**References**
ASD+GI vs. TD	Stool (*n* = 13 and 8) Gastric/SI (*n* = 7 and 4)	16S rRNA sequencing	Phylum, genus, species	Clostridia (g) Ruminococcus (g) Bacteroidetes (p) *Clostridium difficile* (s) Non-sporeforming anaerobes/microaerophiles	↑ ↑ ↑ ↑ ↑	(39)
ASD (*n* = 15) vs. TD (*n* = 8)	Stool	16S rRNA primers RT-PCR	Genus, species	*Clostridium bolteae* (s) Cluster I (~g) Cluster XI (~g)	↑ ↑ ↑	(40)
ASD (*n* = 58) vs. SIB (*n* = 12) vs. TD (*n* = 10)	Stool	FISH – 16s RNA probes	Genus, species	*Clostridium histolyticum* (s)	↑	(41)
ASD + GI (*n* = 33) vs. SIB (*n* = 7) vs. TD (*n* = 8)	Stool	454 Titanium pyrosequencing (culture-ind)	Phylum, genus, species	Firmicutes (p) Actinobacteria (p) Bacteroidetes (p) Proteobacteria (p) Bifidobacterium angulatum (s) *Bifidobacterium longum* (s) Bifidobacterium (g) Bacteroides (g) Clostridium (g) Desulfovibrio (g) Ruminococcus (g) *Desulfovibrio piger* (s), *D. desulfuricans* (s), *D. intestinalis* (s) *Bacteroides vulgates* (s) Overall diversity and richness	↓ ↓ ↑ ↑ ↓ ↓ ↓ ↑ ↑ ↑ ↓ ↑ ↑ ↑	(42)
ASD (*n* = 58) vs. TD (*n* = 39)	Stool	Culture?	Genus, species	Bifidobacterium (g) Enterococcus (g) Lactobacillus (g) Bacillus (g) *Klebsiella oxytoca* (s)	↓ ↓ ↑ ↑	(15)
ASD (*n* = 23) vs. SIB (*n* = 22) vs. TD (*n* = 9)	Stool	16S rRNA primers RT-PCR	Genus, species	Bifidobacteria spp (vs. CON and SIB) *Akkermansia muciniphila* (vs. CON) *Bacteroides fragilis* (ASD: +GI vs. –GI)	↓ ↓ ↑	(21)
ASD + GI (*n* = 15) vs. TD + GI (*n* = 7)	Ileal and cecal biopsies	16S rRNA primers RT-PCR	All levels	Bacteroidetes (p) Firm/Bact (p) Clost/Bact (o) Firm+Prot (p) Clostridiales (o) Lachnospiraceae&Ruminococcus(f) Proteobacteria (p) Betaproteobacteria (c)	↓ ↑ ↑ ↑ ↑ ↑ ↑	(43)
ASD vs. TD (*n* = 20)		454 Titanium pyrosequencing	Phylum, genus	Proteobacteria (p) Verrucomicrobia (p) Akkermansia (g) Prevotella (g) Veillonellaceae (f) Overall diversity	↓ ↓ ↑ ↓ ↓ ↓	(44)
ASD vs. PDD vs. TD (*n* = 10)	Stool	16S rDNA and rRNA primers RT-PCR	All levels	Overall diversity Firmicutes (rRNA and rDNA) (p) Bacteroidetes (p) Fusobacteria and Verrucomicrobia (p) Differential rRNA clustering (not rDNA) Ruminococcaceae (f) Faecalibacterium/Ruminococcus (g) Clostridiaceae (f) Lachnospiraceae (f) Prevotella and Alistipes (g) Sutterellaceae (f) Parasutterella (g) Enterobacteriaceae (f) Proteus and Shigella (g) Bifidobacteria (g) *Akkermansia muciniphila* (s)	↑ ↓ ↑ ↓ ↓ ↑ ↑ ↑ ↑ ↑ ↓ ↑	(45)

*ASD, autism spectrum disorder; TD, typically developing; +GI, with GI symptoms; –GI, without GI symptoms; SIB, sibling of ASD; PDD, pervasive developmental disorder; p, phylum; c, class; o, order; f, family; g, genus; s, species*.

**Table 2:** Overview of studies exploring gut putrefactive metabolites in children with ASDs.

**Comparison**	**Sample site**	**Metabolites**	**Change**	**Microbiota assoc/other comments**	**References**
Infants (*n* = 28), children (*n* = 60), and ASD (*n* = 262)	Urine	HPHPA 3-(3-hydroxyphenyl)-3-hydroxy- propionic acid (Phe metabolite)	↑↑	Clostridia spp (some) hyp Psychosis and level ↓ w antibiotic treatment in schizophrenia	(46)
ASD (*n* = 59) vs. TD (*n* = 59)	Urine	p-cresol Only age <7 and corr with autism severity	↑	*Clostridium difficile* (s) hyp Expresses p-hydroxyphenylacetate decarboxylase	(47)
Case report	Blood/urine	Propionic acid Ammonia	↑ ↑	Associated with pancreatitis	(48)
ASD (*n* = 23) vs. TD (*n* = 31, 22 SIB)	Stool	Ammonia Phenol Acetate, butyrate, propionate Isobutyrate, valerate, isovalerate p-cresol	↑ ↑ ↑ ↑ —	Similar dietary intakes	(49)
ASD vs. PDD vs. TD (*n* = 10)	Stool	Free amino acids (FAA) Ammonia Phenol p-cresol Propionic acid Indole/3-methylindole Total med and short chain fatty acids	↑ ↑ ↑ ↑ ↑ ↑ ↓	↑Bacteroides (g)corr ↑Bacteroides (g)corr ↑Clostridia (g)hyp ↑Clostridia (g)hyp ↑Bacteroides (g) corr ↑Clostridia (g) corr ↓ Ruminococcoceae (f) hyp	(45)
ASD (*n* = 33) vs. TD (*n* = 33)	Urine	Total p-cresol and derivatives p-cresylsulfate p-cresylglucuronate Free p-cresol	↑ ↑ ↑ —	Exclusive to age <8 for all	(50)

*ASD, autism spectrum disorder; TD, typically developing; SIB; sibling of ASD; corr, correlation found; hyp, hypothesis only*.

**Figure 1:**
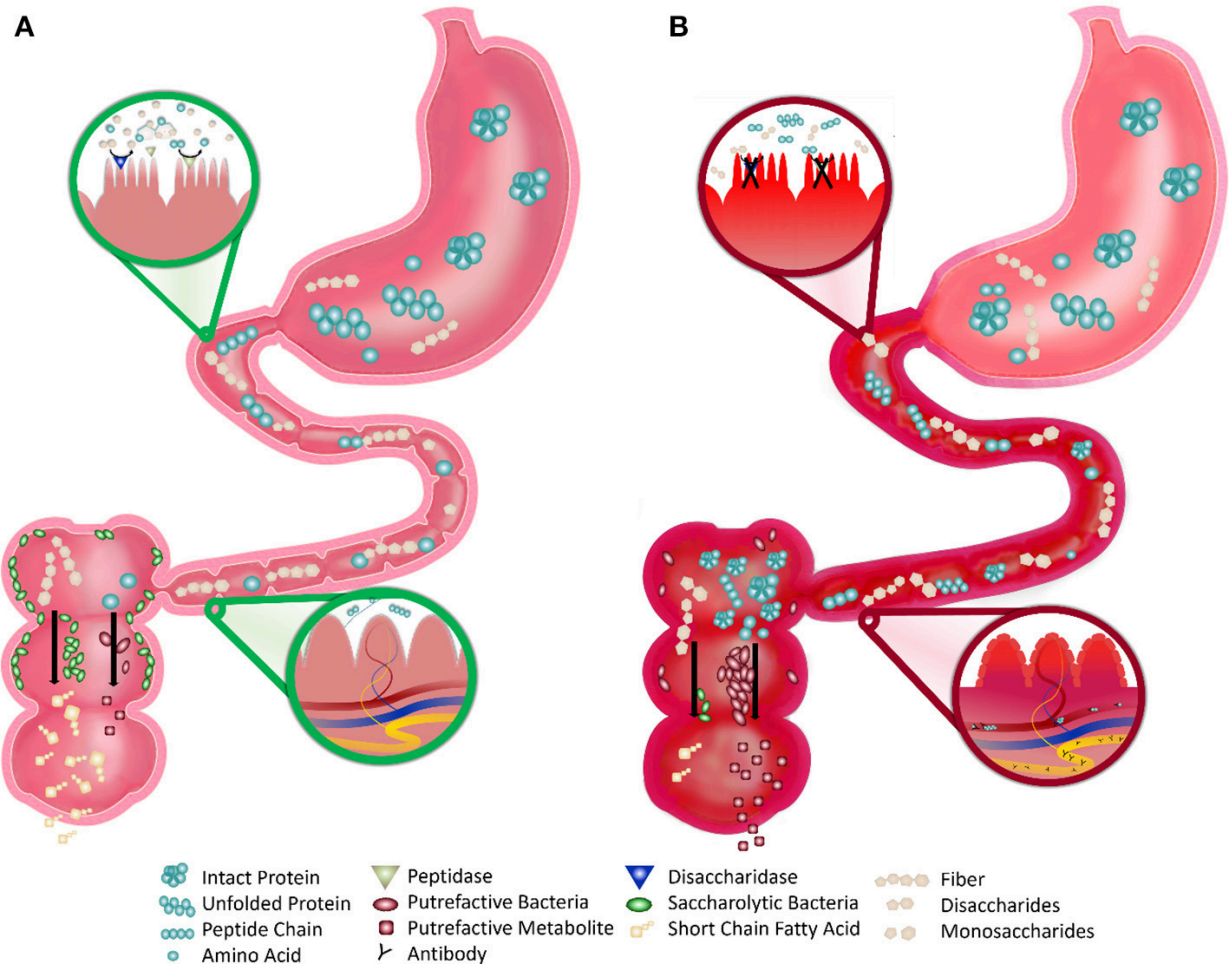
Robust vs. Fragile Gut Function in Children with ASD. **(A)** Robust Gut: the healthy gut displays robust digestion of proteins and simple sugars by the small intestine brush border enzymes that make these nutrients absorbable. After digestion, very few intact nutrients remain and indigestible polysaccharides (fiber) remain. This fiber is consumed by saccharolytic bacteria, which line most of the large intestine, and produce beneficial byproducts (such as short chain fatty acids). Undigested proteins or amino acids are consumed by putrefactive bacteria, which are few in number, and produce potentially harmful putrefactive metabolites that are easily detoxified. The blood and lymphatics in the villi do not directly interact with the lumen of the small intestine, preventing the interaction of antigenic food molecules with the underlying immune tissue. **(B)** Fragile Gut: the fragile gut of children with autism displays reduced digestive capacity. The inflammation and deterioration of the gut lining may cause reduced expression and activity of brush border disaccharidases and peptidases and greater amounts of intact simple sugars and proteinaceous substrates and less fermentable fiber. This proteinaceous substrate is consumed by the more prevalent putrefactive bacteria producing greater amounts of putrefactive metabolites, such as ammonia, phenols, and sulfides. The blood and lymphatics in the villi are in contact with the lumen due to the excessive inflammation and the undigested proteins in the intestine are able to directly pass. This process allows for interaction of antigenic proteins with immune tissue leading to an aberrant immune response and subsequent autoantibody production.

We propose that the fragile gut is influenced by dietary protein digestion. Incomplete digestion increases microbial protein fermentation (putrefaction) and inflammatory putrefactive metabolites, which can result in decreased gut barrier integrity, increased gut-associated immune responses, and microbial population shifts. In some children with ASD, reduced proteolytic capacity and colonic putrefaction may cause gastrointestinal problems and also exacerbate ASD symptoms (Figure 1). Identification of a fragile gut phenotype in ASD has both diagnostic and therapeutic applications. Identification of susceptibility and characterization of exogenous triggers has diagnostic implications. Therapeutic implications include avoidance of specific triggers and enhancement of the “robustness” of the intestinal microbiota, immune system, and intestinal tissues.

It is important to note that most of the available evidence linking gut dysfunction and dysbiosis to ASD is at this point based on associations and not causal relationships. Therefore, we are calling for future experimental and clinical studies designed to specifically address the potential causal relationship between these factors, including the gold standard of dietary intervention trials in children with ASD and GI symptoms.

## Evidence for protein maldigestion in children with ASD

### Low digestive enzyme activity

Protein digestive capacity may be impaired in some children with ASD, which would contribute to the abnormal immune activation and gastrointestinal symptoms observed in these populations (Figure 1). However, there are no studies directly assessing the level and activity of proteolytic digestive enzymes in these children. One study found that the activity of several saccharolytic digestive enzymes is low in children with ASD and gastrointestinal symptoms (51). Characterization of the upper gastrointestinal tract in these children revealed decreased activity of one or more disaccharidases or glucoamylase in 58.3% of children with ASD compared to healthy controls. Remarkably, nearly half of these children showed decreased activity in two or more of these enzymes and all children with low enzyme activity had loose stools and/or gaseousness. Stimulation of pancreatic output with secretin administration showed that children with ASD on average had a higher pancreatico-biliary fluid output compared to healthy controls (51). While secretin treatment is now a disproven therapy (52), the authors hypothesized that the hypersecretory response to intravenous secretin might be due to the absence of regular secretin stimulation of the pancreas. However, this response could also be due to chronic pancreatic insufficiency as increased pancreatico-biliary output has been observed in patients with exocrine pancreatic insufficiency (53). While this study found no evidence of pancreatic insufficiency in children with ASD, they did observe hypertrophy and hyperplasia of the upper gastrointestinal Paneth's cells (specialized immune cells interdigitated between enterocytes of the intestinal epithelium). The authors noted that Paneth cell hypertrophy/hyperplasia has also been reported in hamsters after ligation of the pancreatic duct (54) and in patients with chronic pancreatitis (55).

Later studies investigated brush border enzyme function in children with ASD further. One study found significantly decreased levels of ileal sucrose isomaltase, maltase glucoamylase, and lactase mRNA in children with ASD and gastrointestinal symptoms when compared to typically developing children who also had similar gastrointestinal symptoms (43); therefore, these findings are autism-specific and not generalized to all gastrointestinal dysfunction. Indeed, 93.3% of children with autism in this study had a deficiency in at least one of these enzymes, while 80% had deficiencies in at least two, and 73.3% had deficiencies in all three (with deficiency defined as levels <25% of controls). These digestive enzymes are located on the brush border of the enterocyte membrane, and thus their levels and activity could be decreased by chronic inflammation of the gastrointestinal tract or other disturbances in enterocyte maturation and migration. If this is the case, then other brush border enzymes, such as peptidases, are impaired as well.

### Altered levels of systemic amino acids and peptides

Children with ASD who have shown elevated levels of urinary peptides of dietary origin (56) provide further evidence that this population experiences impaired protein digestion coupled with increased intestinal permeability. Unfortunately, the interpretation of these findings must be viewed with some caution as they have arisen out of the “opioid excess theory.” This theory of ASD's origin hypothesizes that breakdown products of certain dietary proteins (mainly casein and gluten) are potent agonists of opioid receptors that can exert systemic effects and are able to cross the blood brain barrier (56). Indeed, many of the urinary peptides detected from children with ASD can be classified as exorphins (exogenous opioids) and include casomorphins, gliadinomorphins, gluteomorphins, deltorphin and dermorphin. Treatment of these subjects with an extended gluten-free, casein-free (GFCF) diet (2–4 years) resulted in significant reduction in urinary peptide levels as well as improvement in behavioral metrics (57–59), despite abundant intake of meat and fish protein (58). Suggestively, several animal studies have shown that inhibition of gut peptidases, specifically dipeptidyl peptidase IV (DPP-IV), results in increased levels of urinary peptides of dietary origin (60, 61). While the “opioid excess theory” is not widely accepted and supporting evidence is limited, these findings support the hypothesis that a subset of children with ASD experience reduced digestive enzyme activity, and undigested dietary peptides can cross the intestinal mucosal barrier and exert biological effects systemically.

Plasma amino acid profiles tend to be altered in children with ASD, although studies have reported mixed results. While some studies have shown elevated levels of certain plasma amino acids, such as glutamic acid, aspartic acid (62, 63) and taurine (62), others have shown decreased levels of certain amino acids, especially glutamine (62–64). Arnold et al. (65) found that children with ASD on both restricted (GFCF) and unrestricted diets had frequent essential amino acid deficiencies. Compared to controls, children with autism on unrestricted diets were more likely to be deficient in valine, leucine, phenylalanine, and lysine, while children on restricted diets were additionally more likely to be deficient in isoleucine (65). Adams et al. found decreased levels of isoleucine, phenylalanine, tryptophan, and taurine; they hypothesize that this is due to decreased protein intake or impaired protein digestion. They postulated that the latter hypothesis is more likely because most essential amino acid levels were normal (66). Low levels of certain plasma amino acids in children with ASD suggest both an impaired capacity for protein digestion as well as increased passage of dietary peptides into systemic circulation by way of compromised intestinal integrity. Finally, certain plasma amino acids either serve as neurotransmitters (e.g., glutamate) or serve as precursors (e.g., tryptophan and tyrosine) for important neurotransmitters (e.g., serotonin and dopamine). Perturbations in these systems are common in children with autism and contribute significantly to autistic symptoms (67–69).

### Autoantibodies against dietary peptides and gut peptidases

Low proteolytic enzyme activity in the gut would lead to increased levels of undigested peptides in the gut lumen, resulting in increased plasma and urinary concentrations of these peptides. High levels of foreign peptides in the systemic circulation can induce an adaptive immune response that leads to autoantibody (i.e., self-reactive antibody) production (Figure 1). Therefore, the subsequent cross-reaction of the resulting antibodies with neural tissue proteins may play a role in the etiology of autistic symptoms.

Multiple studies have found elevated levels of circulating antibodies against several microbial products including *Chlamydia pneumoniae* peptide *Streptococcus* M protein (70), streptokinase (SK) (71), and heat shock protein (HSP-60) (72) in children with ASD compared to healthy controls. Elevated levels of antibodies against dietary proteins have also been detected in children with autism including IgG, IgM and IgA against butyrophilin milk protein (70), casein (57, 71, 73–75), gliadin (57, 71, 72, 74, 75), IgG against deamidated α-gliadin (75), and IgA against lactalbumin, β-lactoglobulin and ovalbumin (73).

Bacterial proteins and peptides have been reported to show sequence homology and subsequently induce antibody cross-reactivity with certain human brain tissue proteins (74). Immune reactions mounted against dietary peptides may subsequently damage neural tissue in children with ASD that cause or exacerbate behavioral symptoms. Elevated levels of antibodies were found against myelin basic protein and neurofilament proteins in 58-70% of children with ASD (76, 77) with studies corroborating these findings and discovering elevated levels of antibodies against a number of other neuronal proteins including myelin-associated glycoprotein, ganglioside, myelin oligodendrocyte glycoprotein (70), and cerebellar peptides (74). *In vitro* studies have also demonstrated that antibodies against proteins of gut origin are able to cross-react with neuronal proteins and peptides (74). One study showed that antibodies against the gliadin peptide cross-reacted with cerebellar peptide with 17% binding (74). Antibodies against crude (undigested) gliadin showed 23% binding to cerebellar peptide, milk protein showed 56%, egg protein showed 68% binding, while corn or soy protein showed little to no cross-reactivity. Additionally, significant increases in anti-cerebellar peptide antibodies were concomitant with increases in anti-gliadin antibodies in most ASD sera, thus supporting the hypothesis of an autoimmune etiology. Furthermore, studies of the maternal origins of autoantibodies in ASD found that fetal brain protein reactive immunoglobulins can be isolated from approximately 12% of mothers who have children with ASD (78). Subsequent studies using a non-human primate model showed that the administration of these autoantibodies to rhesus monkeys during pregnancy resulted in offspring with abnormal social behavior and enlarged brain volume compared to controls (79). This group has since defined an autism subset, termed maternal autoantibody-related (MAR) autism, defined by the maternal presence of clinically significant ASD-specific antibody biomarkers (80). Children found to fall into the MAR-autism subset experience elevated stereotypical behaviors (80) and more extreme brain enlargement (81) compared to children with other types of autism.

Autoimmune cross-reactivity has also been shown to affect several gastrointestinal proteins in addition to neural proteins. Vojdani et al. hypothesized that antibodies against antigens from infectious agents may react with lymphocyte receptors (71, 74). Two of these lymphocyte receptors, DPP-IV and aminopeptidase N (CD13), are also located on the brush border of the small intestine and are involved in degradation of small peptides as part of the protein digestive process. They found that ASD sera contained significantly greater levels of antibodies against both of these gut peptidases compared to healthy children. In addition, the vast majority of children with ASD who had elevated levels of these anti-peptidase antibodies also had elevated levels of antibodies against gliadin, casein, SK, or HSP-60 and many had elevated levels of all of these antibodies (71, 72). *In vitro* studies found that binding of anti-DPP-IV antibodies to DPP-IV significantly reduced its activity (*p* < 0.05) (72). These findings suggest that children with ASD have elevated levels of antibodies against gut peptidases that may impair their function and lead to increased levels of peptides in the gut. This would increase the likelihood that dietary peptides enter systemic circulation and set off an autoimmune reaction that leads to neuronal protein targeting in the brain.

### Intestinal microbial dysbiosis

Some of the microbial perturbations reported in ASD (Table 1) support the hypothesis of impaired protein digestion in this cohort. Impaired protein digestion would not only result in increased levels of peptides entering the systemic circulation, but also entering the distal gastrointestinal tract. This increased level of proteinaceous substrate could modulate the composition of the microbiota and lead to dysbiosis, an imbalance between protective and detrimental taxa in the gut (Figure 1). While studies on protein fermentation (putrefaction) in the human colon are extremely limited, several studies indicate that increased levels of protein in the colon leads to increased levels of protein-metabolizing (putrefactive) bacteria (82, 83), reduced levels of fiber-fermenting (saccharolytic) bacteria (83–85), and reduced bacterial diversity (85) as well as increased levels of putrefactive metabolites in the colon (83, 85–88). These metabolites have been associated with inflammatory bowel diseases such as ulcerative colitis (89, 90). The effects of these metabolites on host physiology have been studied using mainly *in vitro* models and have been shown to increase intestinal permeability (87, 91–93) and reduce overall health of colonocytes (87, 91, 94–96). Therefore, the prevalence of gastrointestinal symptoms may be directly related to an altered gut microbiota in these children.

Table 1 lists the studies examining the fecal microbiome of children with ASD. Children with ASD appear to have more bacterial diversity (42, 45) and possess lower overall abundance of potentially beneficial taxa, such as *Bifidobacteria* and *Akkermansia*, even among healthy siblings relative to unrelated control children (15, 21, 42), though some populations appear to differ from this trend (42, 44). Further, reports typically show that children with ASD have higher counts of *clostridia* than controls (39–43), though this is not universally true (44). Interestingly, certain species of *clostridia* are very adept at metabolizing protein and amino acids (97–99). Studies examining bacterial enrichment in batch incubations of human fecal slurries supplemented with peptides and amino acids have found an outgrowth of certain species of *clostridia* (97, 99). The majority of the proteolytic activity in the large intestine is attributed to bacterial taxa including *Bacteroides, Clostridium, Propionibacterium, Fusobacterium, Streptococcus*, and *Lactobacillus* (98). Both *Bacteroides* (21, 42) and *Lactobacillus* (15) have been found at higher levels in children with ASD. In addition, rats fed a high protein diet have been shown to have increased levels of *Bacteroides* spp. and certain Proteobacteria, including *escherichia*/*shigella*, as well as decreased levels of *akkermansia* and *bifidobacteria* spp. relative to rats on normal protein diet (83). Proteobacteria have also been shown to be increased in children with ASD (42, 43). These results suggest that proteolytic bacterial taxa are enriched in children with ASD as a consequence of high levels of undigested protein entering the colon. However, studies in this area are limited, and data on the effects of high protein diets on the colonic microbiota in humans are scarce. In addition, the activities of these gut bacteria remain to be determined and future studies will be needed to define the metabolic activities (e.g., metagenomics and metatranscriptomics) of the gut microbiota rather than purely microbial composition.

From a symptomatic perspective, gastrointestinal complaints were significantly associated with *Clostridia* in patients with ASD when compared with healthy unrelated controls (41). In addition to differences in *Bifidobacterium* spp., Wang et al., also found the abundance of *Akkermansia muciniphila* (*A. muciniphilia*) to be decreased in ASD, and again intermediate in siblings, as well as elevated relative numbers of “*B. fragilis”* in children with ASD and gastrointestinal symptoms vs. children with ASD without gastrointestinal symptoms. It was hypothesized that lower levels of *A. muciniphila* indicate a thinner gastrointestinal mucus barrier, since this species is mucolytic and may be reduced due to lack of substrate (21). Williams et al. (43) compared the bacterial adherent to the intestinal mucoepithelium (from biopsies) in children with ASD and gastrointestinal co-morbidities to children with gastrointestinal issues but without ASD. Comorbid conditions were also similar between groups with the most commonly reported conditions being atopic manifestations (asthma, atopic dermatitis, and allergic rhinitis). Bacteroidetes and Firmicutes were the most prevalent taxa in ileal and cecal biopsies of both autistic and control children. However, there was a significant increase in Firmicutes/Bacteroidetes ratios in autistic ileal and cecal samples, which has been interpreted to reflect a general state of dysbiosis in other conditions, such as obesity (100). Overall, these studies support the hypothesis that abnormal microbial composition of the gastrointestinal tract can cause or exacerbate GI symptoms associated with ASD. Other studies suggest that this abnormal microbiota may even be associated with the symptoms and severity of ASD (15). While dietary components are the primary determinants of gut microbial composition after birth in mammals, the cause of the dysbiosis observed in ASD has not been thoroughly studied in the context of diet, protein maldigestion, or gut abnormalities. However, the combined effects of immune imbalance with digestive dysfunction and intake of dietary proteins with low digestibility may all play a role in promoting gastrointestinal symptoms and microbial dysbiosis in this susceptible population. Furthermore, these dysbiotic bacteria may directly cause or exacerbate gastrointestinal symptoms due to their potential to produce damaging metabolic byproducts.

### High levels of fecal putrefactive metabolites

A variety of enteric bacteria, including *clostridia, desulfovibrio*, and Bacteroidetes are enriched in batch cultures high in proteinaceous substrates (97–99) and in children with ASD (39, 42). These bacteria can ferment dietary proteins in a process called putrefaction (98, 101). Byproducts of microbial putrefaction can be detrimental to gut health (102). Microbial metabolites produced from the saccharolytic fermentation of dietary fiber (e.g., vitamins and short chain fatty acids) are generally recognized as being beneficial to human health. In contrast, the products of putrefaction [e.g., ammonia, phenols, indoles, sulfides and biogenic amines (101)] have been associated with reduced viability of colonic epithelial cells *in vitro* (103), increased intestinal permeability (92, 93), DNA damage (104) and the inhibition of cellular respiration in colonocytes (95, 105, 106). Increased colonic protein, due to reduced digestive capacity, serves as a food source for these putrefactive colonic microbes. Thus, a diminished capacity to digest dietary protein in the small intestine and/or consumption of proteins resistant to digestion can lead to elevated colonic protein and peptide levels, and thus increased concentrations of these detrimental protein-derived metabolites. The heightened level of putrefactive metabolites found in children with ASD (Table 2) (47, 49, 63, 107, 108) suggests that protein maldigestion in the small intestine plays a role in driving not only gastrointestinal symptoms, but also neural symptoms of this disorder.

One specific consequence of protein fermentation is the ability of certain gut bacterial species to catabolize threonine and alanine to produce propionic acid, a metabolite that also increases with increased protein in the diet (101). Propionic acid concentrations are higher in children with ASD than healthy children (49). In addition, acute and chronic abnormalities of brain function are well-documented in propionic acidemia, a common form of organic aciduria resulting from an inborn error of metabolism that causes propionyl-CoA carboxylase deficiency (109, 110). One case study reported the occurrence of autism in a 7-year old girl with propionic acidemia (48). While it is possible that these disorders co-occurred by coincidence, the authors postulate that the propionic acidemia contributed to the emergence of ASD symptoms. MacFabe and colleagues have thus eloquently hypothesized that proprionic acid plays a role in the etiology of ASD (107) and have subsequently developed an animal model of ASD using proprionic acid to induce autistic-like symptoms (111). Rats treated intracerebroventricularly with propionic acid displayed numerous behavioral and physiological traits characteristic of human ASD including hyperactivity, repetitive and abnormal motor movements, seizures, increased brain oxidative stress markers (lipid peroxidation products and protein carbonylates), decreased glutathione and glutathione peroxidase activity, and increased neuro-inflammation indicated by increased reactive astrogliosis and activated microglia. Subsequent studies showed that propionic acid-treated rats display social behavior impairments (112, 113), altered brain lipid profiles (114), restricted behavioral interests and cognitive impairment (115), hypophagia and taste avoidance (116), DNA damage (117), as well as sexual dimorphism in regard to acoustic startle response and prepulse inhibition (118), all of which are implicated in human ASD. In addition, another group has shown that clindamycin-induced *clostridia* overgrowth causes propionic acid elevation and subsequent genotoxicity in the brain of hamsters (119), which supports the hypothesis of the gut origin of this metabolite. Propionic acid also affects colonic smooth muscle contraction, mast cell activation, serotonin release, and gastric motility (120); these factors could provide a direct connection between microbial metabolic activity and gastrointestinal symptom occurrence.

Propionic acid is capable of reacting with ammonia, another putrefactive product, to produce β-alanine (121). β-alanine is structurally similar to γ-aminobutyric acid (GABA), an inhibitory neurotransmitter. β-alanine has been found to be higher in children with ASD compared to controls (63), can function as a partial GABA antagonist (122) and can cross the blood brain barrier (123), making it another potential candidate for ASD symptom induction (121). Urea cycle disorders, characterized by hyperammonemia, can also present with confusion, bizarre behaviors, and autistic-like symptoms (124, 125). Finally, several other putrefactive metabolites have also been found to be elevated in the urine and feces of ASD patients including p-cresol (47, 108), p-cresylsulfate, p-cresylglucuronate (108) and ammonia (49). P-cresol and p-cresylsulfate levels were also positively associated with stereotypic and compulsive/repetitive behaviors in these children (108), as well as overall severity of autistic symptoms (47). Elevated levels of putrefactive metabolites have also been found in animal models of ASD including 3-(3-hydroxyphenyl)-3-hydroxypropionic acid, 4-hydroxyhippuric acid, 4-hydroxybenzoic acid (126) and 4-ethylphenylsulfate (127). Abnormally high concentrations of putrefactive metabolites in children with ASD compared to controls could not be explained by dietary intake as consumption of protein, sugar, starch, and fiber was similar between groups (49). These differences may be better explained by digestive capacity, the gut microbiota, and overproduction of putrefactive metabolites in children with ASD, compared to typically developing children.

## Factors affecting protein digestibility

### Inherent properties of proteins

Dietary proteins display a wide range of susceptibility to digestive processes in the human gut depending on sequence, structure and the food matrix in which it is present: certain proteins (e.g., human β-casein) are easily hydrolyzed into amino acids in the small intestine, whereas others (e.g., wheat storage proteins like gliadin and gluten) are highly resistant to proteolysis (128). Protein digestibility depends on several factors, including size, charge, amino acid sequence, tertiary structure (129–131) and post-translational modifications such as glycosylation (132, 133) and phosphorylation (134). In general, even under idealized conditions, proteins from animal sources tend to be more susceptible to enzymatic hydrolysis and demonstrate apparent digestibility over 95% with milk proteins having the highest apparent digestibility at around 98%. Plant proteins have lower apparent digestibility with values ranging from 70 to 85%. However, certain animal structural proteins, such as collagen, elastin, and keratin contain unusual secondary structures that are resistant to proteolysis (128). For example, the collagen triple helix is characterized by stabilizing crosslinks between peptide chains that are difficult to cleave and reduce digestibility. The low digestibility of plant proteins is partially due to similar structural features as well as relative insolubility, compact structures of intracellular protein bodies and high structural integrity of the plant cell wall and seed coat (128). Therefore, vegetable proteins, particularly those found in legumes and cereal grains, require cooking in addition to other processing methods to partially improve digestibility.

Beyond protein structure, dietary protein digestibility can be reduced by anti-nutritional factors that reduce protein bioavailability. These factors include protease inhibitors, tannins and phytates (128) that are commonly found in plant foods (135, 136). Trypsin inhibitors are found in many legumes (common bean, cow pea, lima bean, peanut, garden peas and soy beans) and cereal grains (wheat and barley) as well as potatoes and some squash. These inhibitors bind trypsin, and sometimes chymotrypsin, within the gut lumen to impair protein digestion (128). In addition, many of these anti-proteases are resistant to digestion and are also highly stable to thermal and acid denaturation and so can retain at least partial activity even with cooking (137, 138). Tannins are naturally occurring plant polyphenols that function to bind and precipitate proteins (139, 140) and are found at high levels in legumes, nuts and other minority cereals such as sorghum and barley as well as many types of fruits (136). However, fruit is not a major source of protein in the diet. Tannins have also been shown to reduce feed digestibility by directly inhibit enzymes in the intestines of ruminants (139) and through formation of tannin-nutrient complexes (141, 142). Overall, high levels of tannins in livestock feed diminish weight gains, apparent digestibility and feed utilization efficiency, partially through their action in inhibiting protein digestion (143). Finally, phytates are mineral-binding molecules found in the kernels of cereal grains including wheat, maize, rice, barley, sorghum, oats, rye, and millet (135) that may also decrease protein digestibility (144).

### Protein processing effects

The processing of commodities into foods has important effects on the digestibility of proteins. Both protein structure and anti-nutritional factors can be altered by food processing heat treatments (128, 145). Heat treatment can either increase or decrease protein digestibility depending on the type of protein, and the temperature and duration of treatment (128). For example, cooking increases digestibility of legume proteins, but the increase in temperature negatively affects enzymatic hydrolysis and solubility of sorghum (146, 147) and milk proteins, and negatively affects maize proteins (146, 147). In targeted applications, the effects, of protein thermolyzation on digestibility, have been explored extensively, for example in the context of milk proteins in infant formula, which often undergo extreme heat treatment (148). Interestingly, several studies have shown that extensive heat treatment of milk proteins (e.g., spray drying, in-can sterilization) results in various protein modifications (e.g., increased protein-lipid and protein-protein interactions, increased denaturation, and formation of modified side chains) that reduces their digestibility both *in vitro* (149–153) and *in vivo* (154, 153). Heat treatment of bovine milk to an extent similar to that used in the industrial sterilization and processing of yogurt, makes the casein protein more resistant to digestive processes; this resistance is hypothesized to be due to heat-induced aggregate formation (152). In addition, reaction of the amine functional group on free amino acids with a reducing sugar results in the formation of Maillard reaction products that can also inhibit proteolysis (153). The amino acid lysine is more susceptible to this reaction that other amino acids due to the presence of its ε-amine group. When lysine reacts with the milk sugar lactose, lactosyllysine forms and inhibits the activity of trypsin (153). Other Maillard reaction products were similarly been found to inhibit protein digestion. Furthermore, irradiation, alkaline processing, oxidation, high pressure processing, extrusion and freeze-thaw cycling of foods can also affect protein digestibility (128, 145). In addition to the other deleterious properties of ultra-processed foods [e.g., the presence of harmful additives, preservatives, colorants and other toxins (155)] the negative effects of certain processing techniques on dietary protein digestibility prompt consideration of the exclusion of specific foods and the inclusion of protein sources based on their digestibility by at risk individuals (e.g., minimally processed dairy, eggs, meat, and fish) in the diets of children with ASD.

### Intestinal environment

Beyond the effects of protein structure and processing on digestibility, the individual's digestive environment alters degree of protein digestion. Specifically, individuals vary in their digestive capacity based on the level and activity of luminal proteases and brush border peptidases, gastric and intestinal luminal pH, and microbial proteolytic activity (145). For example, in chronic malnutrition, pancreatic proteases are depleted (156). Pancreatic insufficiency, as determined by reduced fecal elastase-1 levels (<200 μg/g stool), is also common in gastrointestinal disorders such as celiac disease in children (157) and adults (158) that is correlated with the presence of villous atrophy (159) but not nutritional status (160). Other intestinal disorders are also associated with a high incidence of pancreatic insufficiency including irritable bowel syndrome (161), Crohn's disease, ulcerative colitis (162), giardiasis- or cow milk-related enteropathy (163), and diarrhea of infancy (164). Newborn infants are at particularly high risk for pancreatic insufficiency. One study found that 43% of newborn term infants have low elastase-1 levels that reach adult levels by 48 hours of life while preterm infants experience elastase-1 levels <30μg/g that do not reach adult levels until 14 days of life (165). Studies have also found reduced chymotrypsin levels in the stool of prematurely born infants who are small for gestational age compared to prematurely born infants who are appropriately sized for their gestational age (166). Pancreatic insufficiency can also be induced by enteric pathogen colonization leading to acute enteritis in adults (167). In addition, low elastase-1 activity has also been found in diabetics (168) and the elderly (169). Pancreatic insufficiency has also been closely associated with increased markers of gastrointestinal inflammation (170), a common occurrence in children with ASD.

Impaired pancreatic function can be reversed, except for cases of severe and extensive malnutrition in which refeeding does not recover function (171, 172), other cases of pancreatic insufficiency are potentially reversible (163, 169). In the case of celiac disease, the reduced pancreatic exocrine function returns to normal after at least 12 months on a gluten-free diet (163). In addition, the symptoms of pancreatic insufficiency can be improved with enzyme supplementation (169). These observations suggest that it is possible to ameliorate digestive deficiency through enzyme supplementation as well as through implementation of personalized diets for individuals with reduced digestive capacity. For the fragile gut of children with ASD, this means a nutrient-rich diet containing appropriate protein sources.

The ability to routinely diagnose digestive issues is limited. The gold standard for assessment of pancreatic insufficiency (elastase-1 levels below 200μg/g) is an indirect measure of pancreatic exocrine function (173). One study (174) compared the fecal elastase-1 test with a direct assessment of pancreatic function, the pancreatic function test (PFT) (174). Results demonstrated that the correlation between the pancreatic function test and elastase-1 results were poor. The elastase-1 test has a sensitivity of only 41.7%, a specificity of 49.2% and a positive predictive value of only 14% (174). Thus, the incidence of pancreatic insufficiency is underestimated and the development of more sensitive indirect assessment tools is warranted. Furthermore, exocrine pancreatic function is only one dimension of protein digestive capacity and impairments in other aspects of this process are beginning to be explored (145).

## Links between gut health and behavior

It is now recognized that there is a connection between the health of the gut and the presence of psychiatric symptoms. For example, irritable bowel syndrome is associated with a high prevalence of depression, panic disorder, generalized anxiety and post-traumatic stress disorder (175). Explorations into the sources of these co-morbidities have led to hypotheses concerning the composition and activity of the gut microbiota and their interaction with the host immune system (176). There are several mechanisms by which the intestinal microbiota modulate the function of the central nervous system to influence mood and behavior including: augmentation of gut barrier integrity with subsequent changes in systemic levels of bacterial components and metabolites; synthesis of neuropeptides and other neurotransmitters; modification of local and systemic inflammation; moderation of absorbed nutrients, such as decreasing absorption of beneficial and essential nutrients (e.g., vitamins, short chain fatty acids, essential fatty acids, essential amino acids) while concomitantly increasing synthesis of detrimental compounds (e.g., putrefactive metabolites mentioned earlier); modulation of brain-derived neurotrophic factor; and regulation of microbial proliferation by increasing small bacterial overgrowth and/or gastric/intestinal pathogens (12). Thus, the composition and activity of the gut microbiota during critical periods of development has a profound influence on both the development of immune function and the development of the central nervous system in children with ASD as well. However, causation cannot be implied based on this observation. Treatment of children with ASD with oral vancomycin (an antibiotic not readily absorbed by the gastrointestinal tract and thus limited in its effect to the gut lumen) has a short-term benefit for some symptoms of autism in some individuals (177), which is expected if certain microbial pathogens are contributing to behavioral symptoms. Therefore, the presence of gut microbial dysbiosis during critical periods of development has an impact on both immune and neurodevelopment. Early dietary considerations must be taken into account to promote the proper development of these systems.

## Efficacy of restricted diets

Given the general detrimental effect that consumption of proteins with low digestibility could have in children with ASD, examination of studies that consider diets where several of these proteins are eliminated can provide insight into the hypothesis of impaired protein digestive capacity in this cohort even when specific mechanisms of action have not been determined. Several observational and clinical studies looking at the effect of specific protein-free diets, such as GFCF, have reported mixed results in regard to benefit in reduction of gastrointestinal symptoms and behavioral problems (178). Multiple studies have reported significant beneficial effects of the GFCF diet including normalization of urinary peptide levels (179), reduced intestinal permeability (25), and overall improvement of behavioral metrics (179–183). ASD children with gastrointestinal disturbances have been found to show greater improvement in ASD behaviors (e.g., self-stimulatory behaviors, hyperactivity, sensory seeking behaviors, temper tantrums, lining up objects, and echolalia), physiological symptoms (e.g., bodily rash, red ring around anus, constipation, diarrhea and seizures), and social behaviors (e.g., social responsiveness, eye contact, engagement, attention span, requesting behavior, naming objects, pointing, language production, and imaginative play) on the GFCF diet compared to children with ASD but without overt gastrointestinal symptoms (183).

There are important limitations to all dietary intervention studies conducted in this field, such as short duration of treatment (<1 year), heterogeneous sampling (did not select for patients with gastrointestinal symptoms), lack of reporting of changes in gastrointestinal symptoms, and lack of testing for compliance, which is important considering that these proteins are ubiquitous in the modern diet. Indeed, strict implementation of the diet has resulted in substantially greater improvement ASD behaviors, physiological symptoms, and social behaviors (183). In addition, there are likely other dietary proteins that are similarly difficult to digest that have not been considered and need to be minimized in cases of a fragile gut. Overall, dietary intervention studies in these children have not been able to address the multiple, integrated consequences of inappropriate proteins in the diets of individuals with ASD, including dietary protein digestion, gut-associated immune responses, and potential perturbations to the intestinal microbiota, much less provide the diagnostics to build tailored solutions. The future of research in this area will require investigation of whole food components, as gluten-free/casein-free diets are actually wheat-free/dairy-free, and thus food needs to be the focus rather than isolated dietary components.

## Factors affecting food choice affect protein consumption in children with autism

Children with ASD are notoriously “picky eaters”; they have particularly strong food aversions and can be ultrasensitive to food context, especially texture (184–186). Specifically, this cohort has a high oral sensitivity level (184, 187), which means that the children will choose, crave, and avoid certain food smells, tastes, temperatures, and textures (184). For example, it has been observed that children with ASD are less likely to be able to identify sour or bitter tastes and have a decreased olfactory sense compared to typically developing children (188).

Along with an oral sensitivity, children with ASD have lower facial feedback when performing pleasurable tasks that induce laughter or other positive reactions normally seen in typically developing children (189). This decreased facial feedback could have an impact on the way that these children consume foods. One research study proposed that texture preference is based on the sensation received from mouth movements, such as whether the food is chewed, crunched, smushed, or sucked (190).

Sensorial properties of foods are important, especially in children with ASD, since proteins affect food texture and are not homogenous in the diet. Different food commodities provide various types of proteins with varying configurations, including differences in amino acid composition as well as secondary and tertiary structure. Some dietary proteins are easily broken down completely into their constitutive amino acids during digestion in the small intestine, whereas others are more resistant to digestion (128). These proteins can survive intact or partially intact to the colon where they serve as a food source for putrefactive bacteria (191). However, consumers typically select and ingest proteins not on the basis of digestibility, but rather on their sensorial properties.

Palatability varies and is dependent upon food processing methods that alter protein texture and structure. For instance, there are many industrial meat processing techniques that change the texture and the palatability of the meat such as, the use of a pulsed electric field that perturbs cellular architecture (192), the preparation of meat batter at high temperatures that denatures myosin and myofibrillar proteins (193), and the adjustment of pH that alters meat tenderization properties (194). Additionally, dairy product treatments affect palatability by changing the structures of proteins and lipids. For example, sonication of dairy products disrupts protein composition (195), and homogenization and heat treatment cause increased protein-lipid interaction and disruption of milk fat globule structure (153). Therefore, processing of meat and dairy products could alter chemical structures in these foods and may reduce palatability for groups particularly sensitive to food texture, such as children with ASD.

The necessity to implement personalized diets based on an assessment of individual responses to different protein sources in this population is crucial given this populations' tendency toward food aversions, preferences and dietary needs. Occasionally, children with ASD consume inadequate amounts of protein. While there is limited research on differences in protein intake in affected children, one study found that 42.1% of autistic children consume inadequate amounts of proteins (defined as <77% of the daily recommended intake) (186). While other studies have shown no differences in intake level (196), some have shown decreased protein consumption in ASD children compared to other family members (197). In addition, protein-containing foods were least preferred by the children with ASD as 67.4 ±18.7% of the children studied were reported to “never consume” them. Comparatively, foods high in starches were most preferred as only 44.2 ± 22% of the children with ASD never consumed them (186). However, these studies are extremely limited and protein source/processing has not been considered. In another study, children with autism were reported to consume significantly fewer foods in dairy (*p* = 0.001) and protein (*p* = 0.001) categories than typically developing children (198). In addition, there was no difference in the number of foods consumed from each food category by families which suggests it was not the family's eating habits having the effect (198). In view of this review's hypothesis, if children have an immune reaction to certain dietary proteins, they would develop aversions to foods high in protein. Neural pathways from the brain may be altered in response to the immune activation from indigestible proteins in the fragile gut (199). If indigestible proteins are a contributing cause of inflammation, then a personalized diet that avoids specific proteins would be beneficial. Future advancements in the development of personalized diets will need to look for diagnostic tools capable of determining the effects of specific protein consumption in this cohort. To ensure compliance, the organoleptic and sensorial properties of foods and the taste preferences of the ASD individual into account. Well-annotated databases of nutritional requirements and taste preferences need to be integrated in order to design personalized diets.

## Potential treatment options

### Probiotic supplementation

Probiotics, as defined by the WHO in 2001 (200), are “live micro-organisms which, when administered in adequate amounts, confer a health benefit on the host.” They are often, but not always, lactic-acid producing bacteria, such as *lactobacilli, lactococci*, and *bifidobacteria*, or yeasts such as “*saccharomyces boulardii”* (201). While probiotic therapy has been suggested in multiple reviews as a potential treatment for children with ASD and gastrointestinal symptoms (1), there have been no randomized controlled trials of this type of intervention in this cohort to date. Even so, it has been reported that as of 2009, one-fifth of physicians encourages use of probiotics for children with ASD (202). One study showed that *Lactobacillus acidophilus* administration (5 × 10^9^ CFU/day, twice a day for 2 months) significantly reduced a marker of invasive pathogenic candidiasis in children with ASD and gastrointestinal symptoms (203). However, half of the children in the study were on restricted diets and the study design lacked a control group. A separate observational study demonstrated reduced levels of myeloperoxidase, a marker of inflammation and oxidation, in children with ASD and gastrointestinal symptoms who were undergoing probiotic therapy compared to similar children not taking probiotics (204). Probiotic supplementation has been used in animal models of autism with efficacy. Hsiao et al. (127) demonstrated gastrointestinal barrier defects and microbiota abnormalities in a maternal immune activation (MIA) mouse model of ASD. Oral treatment for offspring with MIA with the human probiotic “*Bacteroides fragilis” (“B. fragilis”)* not only increased gut barrier integrity and reversed some microbial alterations, but also ameliorated certain behavioral deficits. Defects in communicative, stereotypic, anxiety-like and sensorimotor behaviors were significantly improved with probiotic administration. Therefore, further investigation into the role of probiotic supplementation in children with ASD and gastrointestinal symptoms via properly designed randomized controlled trials that correlate behavioral and physiological outcomes is warranted.

### Digestible proteins

A treatment option for children with ASD and gastrointestinal symptoms is to replace indigestible proteins in the diet with more digestible sources. While GFCF, diets (which are actually wheat-free/dairy-free) have shown some efficacy in reducing ASD associated gastrointestinal co-morbidities, as previously mentioned, the effect is often marginal with some studies showing greater benefit than others (25, 178–183). Ambiguity in outcomes may in part be due to the simple fact that these dietary protein sources are replaced by alternative protein sources that are also indigestible, such as maize, legumes and other plant-based proteins. In addition, these gluten-free options also tend to be high in refined carbohydrates and also promote bacterial dysbiosis in the fragile ASD gut. In order to increase the efficacy of the GFCF diet, protein sources need to be replaced by highly digestible protein. Many researchers and clinicians hesitate to recommend restricted diets for children with ASD due to dietary imbalances, sensory preferences and low protein intakes that may result from this recommendation (205). However, a properly designed diet that includes digestible protein sources, as well as recommended amounts of vitamins and minerals, can be implemented to improve gastrointestinal function and nutritional status in children with ASD. Combination treatments that utilize high quality protein diets and appropriate complex carbohydrate intake, would promote beneficial bacterial colonization, increase saccharolytic fermentation, reduce microbial putrefaction and subsequently normalize gut inflammation and permeability. This improved gut function could result in reduced ASD symptoms that are a consequence of circulating putrefactive and inflammatory bacterial metabolites as well as diet-derived autoantibodies on nervous system function in children with ASD and gastrointestinal co-morbidities.

### Digestive enzymes

If digestive capacity is impaired in a subset of children with ASD, another potential treatment option is digestive enzyme supplementation, specifically with a full panel of proteases. A limited number of studies have been conducted to test the efficacy of digestive enzyme supplementation on gastrointestinal and behavioral outcomes in children with ASD. One study (206) utilized a unique combination of proteases called ENZYMAID that included alpha-fetoprotein (AFP), bromelain, CASE-GLUTINASE, and phytase (206). AFP is acid-stable and breaks up large proteins into peptides in the stomach, bromelain is a combination of proteolytic enzymes found in pineapple, CASE-GLUTINASE contains both exo- and endopeptidases and has DPP-IV activity, and phytase acts to break down phytate (a plant mineral-binding protein) in order to increase mineral bioavailability. Significant improvement in all parameters measured was observed including eye contact, attention, mood, anxiety/compulsion, comprehension, digestion, and sleep. The most significant improvements were observed in socialization (90% improved) and hyperactivity (80% improved) (206). A more recent study corroborated these findings using Neo-Digestion oral solution containing papain and pepsin (207). Results found significant improvements in emotional response, general autistic impression scores, general behavior and gastrointestinal symptoms including stool quality, abdominal pain, vomiting and food variety. The main behavioral improvements included less restricted and repetitive behavior as well as stereotypic behavior. No correlation was observed between behavior improvement and gastrointestinal symptom improvement, suggesting that behaviors did not improve simply due to lessening of gastrointestinal symptoms (207). While another study found no benefit of enzyme supplementation, this study did not control for probiotic use, which was noted for some subjects (208). Previous studies have shown that some probiotic products contain bacterial strains with potent DPP-IV activity (206), which could confound the results.

## Summary and conclusions

In conclusion, impaired gut immune and digestive function, high levels of circulating dietary peptides, presence of autoantibodies that cross react with dietary proteins, increased levels of putrefactive metabolites with potent actions on gut and behavior, and the microbial patterns associated with ASD, such as high prevalence of clostridial species and high ratios of Firmicutes to Bacteroidetes, suggest that protein-driven microbial dysbiosis exacerbates both gastrointestinal and ASD symptoms. In general, saccharolytic microbes that thrive off of dietary glycans (such as fermentable fibers) tend to produce short chain fatty acids, lower the pH of the intestine, lower pathogens and promote the maturation and metabolism of gut epithelial cells. In contrast, proteolytic microbes that thrive off of incompletely digested dietary proteins produce byproducts such as ammonia, amines, phenols, and sulfides that have a negative effect on gut health (102) and brain function (120). Therefore, the consumption of dietary proteins that are difficult to digest by the reduced digestive capacity of the fragile ASD intestine results in elevated levels of peptides in the gut. These aberrant peptides have the potential to negatively affect gut barrier integrity, feed proteolytic bacteria that produce harmful byproducts, and set off an aberrant immune response, either directly or through the promotion of bacterial dysbiosis. Microbial dysbiosis can promote further improper activation of the immune system leading to a vicious cycle of dysbiosis, inflammation and further damage to gastrointestinal tissue and function. The working hypothesis is that children with ASD experience reduced proteolytic capacity that leads to colonic putrefaction that causes gastrointestinal problems that exacerbate ASD symptoms (Figure 1). Therefore, characterizing the digestive capacity of children with ASD is a personalization strategy capable of guiding diets to include appropriate source and processing methods for dietary proteins. This style of personal intervention will allow for the design and implementation of individualized diets, with particular consideration of protein source and processing, to improve gastrointestinal function and promote a beneficial microbial composition in children with ASD.

Understanding the gut etiology of disease has been hindered by technological and clinical limitations that have inhibited advancements in the field of dietary protein digestion. The application of peptidomics will allow for characterization of protein digestion site, specifically within the gastrointestinal tract. Currently, protein digestion is viewed as a single event definable in very narrow terms when, in fact, digestion is a very broad process varying in multiple aspects of space and time. Recent technological advancements in the area of proteomics and peptidomics now provide the capabilities to perform extensive characterization of digestive processes occurring continually along the length of the small intestine (209–212). Likewise, the technologies to map protein digestion in the gut—peptidomics—is only recently emerging (213, 211). The development of innovations for sampling the human gut, the progression of improvements in peptidomics, and the application of biomarkers for protein digestion (including metabolomics, microbial sequencing and multiplexed inflammatory protein monitoring), will enhance understanding in this field. Specifically, these innovations will improve knowledge of dietary proteins' digestive processes in specific sections of the gut in various populations. Intestinal protein digestion can affect regulatory processes, immune development, and microbial colonization, especially during the perinatal period, and in other susceptible populations where inadequate digestion is particularly harmful.

Further research in this area should expand on the current body of literature that has focused on determining associations between gut function and ASD symptoms to include experimental and clinical studies designed to determine causal relationships between these factors. These studies will have broad implications to many diseases associated with gastrointestinal function, and knowledge gained will play a crucial role in the design of personalized diets with protein sources tailored to individuals with a fragile gut.

## Author contributions

MS wrote the manuscript based on her original ideas and designed the figure. JK wrote sections of the manuscript and created the figure. KA contributed to the overall concept of the manuscript and provided content expertise on ASD and behavioral symptoms, and JG revised the work critically for important intellectual content and provided approval for publication of the content. All authors have read and approved the final version of the manuscript.

### Conflict of interest statement

JB German is a cofounder of Evolve Biosystems a commercial entity providing products for breast fed infants. The other authors declare that the research was conducted in the absence of any commercial or financial relationships that could be construed as a potential conflict of interest.
